# Bioactive Terpenoids and Flavonoids from *Daucus littoralis* Smith subsp. *hyrcanicus* Rech.f, an Endemic Species of Iran

**DOI:** 10.1186/2008-2231-22-12

**Published:** 2014-01-07

**Authors:** Fatemeh Yousefbeyk, Ahmad Reza Gohari, Zeinabsadat Hashemighahderijani, Sayed Nasser Ostad, Mohamad Hossein Salehi Sourmaghi, Mohsen Amini, Fereshteh Golfakhrabadi, Hossein Jamalifar, Gholamreza Amin

**Affiliations:** 1 Department of Pharmacognosy, Faculty of Pharmacy and Medicinal Plants Research Centre, Tehran University of Medical Sciences, Tehran 14155-6451, Iran; 2 Department of Toxicology, Pharmacology and Nanotechnology Research Centre, Tehran University of Medical Sciences, Tehran 14155-6451, Iran; 3 Department of Medicinal Chemistry, Faculty of Pharmacy, Tehran University of Medical Sciences, Tehran 14155-6451, Iran; 4 Department of Drug and Food Control, Faculty of Pharmacy and Pharmaceutical Quality Assurance Research Centre, Tehran University of Medical Sciences, Tehran 14155-6451, Iran

**Keywords:** *Daucus littoralis* Smith subsp. *hyrcanicus* Rech.f, Antioxidant, Cytotoxic activity, Antimicrobial

## Abstract

**Background:**

*Daucus littoralis* Smith subsp. *hyrcanicus* Rech.f. (Apiaceae) is an endemic species in northern parts of Iran where it is commonly named Caspian carrot. The fruits have been used as condiment.

**Methods:**

In a series of *in vitro* assays, antioxidant (DPPH and FRAP assays), cytotoxic and antimicrobial activities of different extracts of roots and fruits were evaluated for the first time. The separation and purification of the compounds were carried out on the most potent extracts using various chromatographic methods and identified by spectroscopic data (^1^H and ^13^C NMR).

**Results:**

The results showed that among the extracts only fruit methanol extract (FME) has significant antioxidant activity (IC_50_ = 145.93 μg.ml^-1^ in DPPH assay and 358 ± 0.02 mmol FeII/g dry extract in FRAP assay). The radical scavenging activity of FME at 400 μg.ml^-1^ was comparable with *α*-tocopherol (40 μg.ml^-1^) and with BHA (100 μg.ml^-1^) (*p* > 0.05). FME did not show any toxicity against cancerous and normal cell lines. Fruit ethyl acetate extract (FEE) had cytotoxic activity against breast carcinoma and hepatocellular carcinoma cells (IC_50_ 168.4 and 185 μg.ml^-1^, respectively), while it did not possess antioxidant activity in comparison with α-tocopherol and BHA as standard compounds. Ethyl acetate and methanol extract of fruits showed antimicrobial activity against *Staphylococcus aureus* (MIC: 3.75 mg.ml^-1^) and *Candida albicans* (MIC: 15.6 and 7.8 mg.ml^-1^, respectively). Four terpenoids were isolated form FEE including: *β-*sitosterol (1), stigmasterol (2), caryophyllene oxide (3), *β*-amyrin (4). Also, three flavonoids namely quercetin 3-O-*β*-glucoside (5), quercetin 3-O-*β-*galactoside (6) and luteolin (7) were isolated from FME.

**Conclusion:**

This study showed that FEE and FME of *D. littoralis* Smith subsp. *hyrcanicus* Rech.f. had the highest biological activities which may be correlated with *in vitro* cytotoxic, antimicrobial and antioxidant activities of terpenoids and flavonoids components of the extracts.

## Background

*Daucus littoralis* Smith subsp. *hyrcanicus* Rech.f. (Umbelliferae or Apiaceae) is an endemic species which is distributed in north of Iran (Mazandaran and Guilan provinces). It is an annual or perennial herb growing up to 3 to 10 cm high on the sandy dunes of Caspian Sea coasts where the fruits have been used as condiment by the rural population [1]. The fruits of the related species, *D. carota*, have been used in Traditional Chinese Medicine (TCM) as a remedy for the treatment of ancylostomiasis, dropsy, chronic kidney diseases and bladder afflictions [2]. A wide range of pharmacologic properties such as antibacterial, antifungal, anthelmintic, hepatoprotective and cytotoxic activities have been reported on *D. carota*[2]. Phytochemical studies indicated the presence of sesquiterpenes, chromones, flavonoids, coumarins and anthocyanins from *D. carota*[2], sesquiterpene lactone [3] and phenylpropanoid triesters [4] from *D. glaber* (Forssk.) Thell. Recently we reported the composition and antimicrobial activity of the essential oil from leaves and stems, fruits, flowers and roots of *D. littoralis* Smith subsp. *hyrcanicus* Rech.f. [5]. No data on the phytochemistry and biological activity have been published for this species. In this study, we investigated the antioxidant, antimicrobial and cytotoxic activities of different extracts from roots and fruits of this plant. Also the isolation and structure elucidation of active compounds from most active extracts are reported.

## Methods

### General procedures

^1^H and ^13^C-NMR spectra was acquired using a Bruker Avance TM500 DRX (500 MHz for ^1^H and 125 MHz for ^13^C) spectrometer with tetramethylsilane as an internal standard, and chemical shifts are given in δ (ppm). Column chromatography was performed using silica gel (70–230, 230–400 mesh) (Merck, Germany) and Sephadex LH_20_ (Fluka,Switzerland). Silica gel 60 F254 precoated plates (Merck, Germany) were used for TLC. The spots were detected by spraying anisaldehyde-H_2_SO_4_ (Sigma-Aldrich Chemie, Germany) reagent followed by heating. HPLC separations were carried out on a Knauer system (Smart line system, Germany) connected to a photodiode array detector. All the solvents, standards and reagents were obtained from Merck (Germany).

### Plant material

The plant was collected from Bandar-e-Anzaly sea coast, province of Guilan, north of Iran, during the fruiting stage in June 2012. A voucher specimen of plant (6734-TEH) was deposited in Herbarium of Department of Pharmacognosy, Faculty of Pharmacy, Tehran University of Medical Sciences, Tehran, Iran.

### Extraction and Isolation

The roots and fruits of plant (1 kg, each) were powdered and extracted successively with ethyl acetate, methanol and methanol–water (1:1), at room temperature. The fruit ethyl acetate extract (FEE) (88 g) was subjected to silica gel column chromatography (CC) with CHCl_3_: AcOEt (9: 1) as eluent to give ten fractions (A-J). The fraction H (5 g) was submitted to silica gel CC with hexane: AcOEt (8: 2) to obtain 20 fractions (Ha- Ht). The fractions Hm and Hr result compounds 1 and 2 (5 and 5.3 mg). The fraction Hc (300 mg) was subjected to CC with hexane: AcOEt (9: 1) to give three fractions (Hc_1_-Hc_3_). Compound 3 (5.9 mg) was obtained from the fraction Hc_3_ (25 mg) by silica gel CC and hexane: CHCl_3_: AcOEt (18: 1: 1) as mobile phase.

The fraction Hd (500 mg) was chromatographed on Sephadex LH_20_ with CHCl_3_: MeOH (3:7) to obtain nine fractions (Hd_1_-Hd_9_). Fraction Hd_9_ (30 mg) was subjected to normal phase semi-HPLC a Eurospher column (250 × 18 mm i.d.) and a PDF detector (λ: 210 nm).The initial eluted ratio was adjusted with 95: 5 (hexane: AcOEt) and delivered to the column for 20 min (flow-rate: 3 ml. min^-1^). Then the eluted ratio was changed to 85:15 (hexane: AcOEt) until 50 min. The program was continued with the same ratio of solvents for next 20 min (70 min after starting point). The compound 4 (5.5 mg) was purified with this method.

The FME was dissolved in distilled water and after filtration; the aqueous solution was extracted with petroleum ether three times. The water soluble phase was evaporated to dryness and then extracted with n-BuOH for three times. The butanolic extract (8 g) was subjected to column chromatography on Sephadex LH_20_ with MeOH to obtain 7 fractions (B_1_-B_7_). Fraction B_6_ was submitted to reversed phase semi- HPLC including a Eurospher (column 250 × 20 mm i.d.) and a PDA detector (λ: 310 nm). Mobile phase including 40:60 (H_2_O: MeOH) was delivered at flow rate 3 ml.min^-1^ to give compounds 5 and 6 (5.5 and 5 mg respectively).

For purification of fraction B_7_, a gradient reversed phase semi-HPLC was used with the same column, flow rate and detection condition. The eluted ratio was adjusted with 65:35 (H_2_O: MeOH) as starting ratio and delivered to the column for 50 min and then was changed to 9:11 (H_2_O: MeOH) until 70 min. Chromatography was continued with the same ratio of mobile phase for next 100 min (170 min after starting point) to give compound 7 (6.5 mg).

### Antimicrobial activity of extracts

Antimicrobial activity of different extracts were tested against a Gram-positive (*Staphylococcus aureus* ATCC 6538), two Gram-negatives (*Escherichia coli* ATCC 8739 and *Pseudomonas aeruginosa* ATCC 9027) and a fungal strain (*Candida albicans* ATCC 1023). Minimum inhibitory concentration (MIC) of the extracts was determined by broth micro dilution method using 96 U-shaped wells plates [6]. A stock solution of 300 mg.ml^-1^ from each extract was prepared in DMSO. Then two-fold serial dilution of the stock solution of each extract (100 μl) was prepared by using Mueller Hinton Broth (MHB) and Sabourad Dextrose Broth (SDB) (100 μl, each) in ten wells. The stock microbial suspension with twofold test inoculum was prepared in MHB and SDB from a 24-h old culture. Then aliquot of 100 μl of twofold test strain inoculum was added to each well to reach the final inoculum size of 5 × 10^5^ cfu.ml^-1^[7]. The minimum bactericidal concentration (MBC) was determined by quantitative subculture of 100 μl from each clear well onto Mueller Hinton Agar (MHA) and Sabourad Dextrose Agar (SDA) plates. Plates were incubated at 37°C and 20-25°C for bacterial and fungal strains, for 48 h, respectively. The MBC is defined as the lowest concentration of extracts that results in more than 99.9% killing of the bacteria being tested [7].

### Antioxidant activity

#### DPPH radical-scavenging activity assay

The antioxidant activity of extracts were measured by the DPPH (2, 2′-diphenyl-1-picrylhydrazyl) free radical scavenging method based on an established protocol [8]. Sample solutions (1 ml) in methanol at different concentration were added to DPPH methanol solution (2 ml, 40 μg.ml^-1^). The mixtures were incubated at room temperature for 30 min and the absorbance was measured at 517 nm. Vitamin E and butyl hydroxyanisole (BHA) were used as positive controls. IC_50_ values (indicate the concentration of the test samples providing 50% radical scavenging) were calculated from graph-plotted scavenging percentage against extract concentration.

#### Ferric reducing antioxidant potential (FRAP scavenging) assay

The FRAP assay was done according to the method described by Benzie and Strain [9,10]. Briefly, the FRAP reagent contained 5 ml of a (10 mmol.l^-1^) TPTZ (2, 4, 6- tripyridyl- s- triazine) solution in 40 mmol.l^-1^ HCl plus 5 ml of (20 mmol.l^-1^) FeCl_3_ and 50 ml of (0.3 mmol.l^-1^) acetate buffer, pH 3.6 and was prepared freshly. Aliquots of extract (50 μl) were mixed with FRAP reagent (1.5 ml), incubated at 37°C, for 10 min, and then the absorbance was measured at 593 nm. For construction of calibration curve, five concentrations of FeSO_4_ .7H_2_O (125, 250, 500, 750, 1000 mmol.l^-1^) were used. The antioxidant activities were expressed as the concentration of antioxidants having a reducing ability equivalent for 1 mmol.l^-1^ FeSO_4_[11].

### Measurement of total phenolic contents

Total phenolics were determined colorimetrically by the Folin-Ciocalteu method as described by Miliauskas, et al. [12]. The prepared extracts (1 ml) were mixed with 5 ml of Folin-Ciocalteu reagent (previously diluted tenfold with distilled water) and allowed to stand at room temperature for 10 min. A 4 ml sodium bicarbonate solution (75 g.l^-1^) was added to the mixture. After 30 min at room temperature, absorbance was measured at 765 nm using a UV spectrophotometer (Pharmacia Biotech). Total phenolics were quantified by calibration curve obtained from measuring the absorbance of a known concentration of gallic acid (GA) standard (20–200 mg.l^-1^). The concentrations are expressed as milligrams of gallic acid equivalents (GA) per g dry extract [11].

### Cell cultures and cytotoxicity assay

Three cancerous cell lines HT29 (colon carcinoma), HepG2 (hepatocellular carcinoma), MCF7 (breast ductal carcinoma) and a normal cell line NIH-3T3 (Swiss mouse embryo fibroblast) were purchased from the Pasteur Institute, Tehran, Iran. The cells were maintained in RPMI 1640, supplemented with 10% fetal bovine serum, 0.28 units.ml^-1^ insulin, 100 μg.ml^-1^ streptomycin, 100 units.ml^-1^ penicillin, and 0.3 mg.ml^-1^ glutamine. The cells were grown at 37°C in a humidified atmosphere of 5% CO_2_. The cytotoxicity of different extracts was assayed using the MTT cytotoxicity assay. The cells (1 × 10^4^) were plated in 100 μl of medium/well in 96-well plates (NUNC, Denmark). After 48 hours incubation at 37°C, in 5% CO_2_, and a humidified atmosphere, the different extracts were added to the cells of different concentrations (800, 400, 200, 100, 50, 25, 12.5 and 6, 25 μg.ml^-1^). Methotrexate (positive control) and extracts were incubated at 37°C, in 5% CO_2_, humidified atmosphere, for 48 hours. After 48 hours, 25 μl of 5 mg.ml^-1^ MTT (dissolved in PBS) was added per well. After three hours of incubation, the MTT solution was removed and the cells were washed twice with 100 μl of PBS. One hundred and fifty microliters of DMSO was added per well, to solubilize the formazan crystals. The optical densities of the wells were then measured at 570 nm (690 nm reference wavelength). By referring to the control (medium with DMSO), the cell survival was assessed [13]. The median growth inhibitory concentration (IC_50_ values) was obtained from the IC_50_ of dose response curve in the Sigma Plot 12 software. Each data is the mean value of three independent experiments and presented as mean ± SD.

## Results and discussion

In the present study extracts from roots and fruits of *D. littoralis* Smith subsp. *hyrcanicus* Rech.f. were investigated for bioactivity, the first time. Among the tested extracts, fruits methanol extract (FME) and fruit ethyl acetate extract (FEE) showed highest bioactive properties. As shown in Table 1, FME had the highest content of total phenol (99.1 ± 0.08 mg gallic acid equivalent/g dry extract) and the highest antioxidant activity in the DPPH assay (IC_50_ = 145.93 μg.ml^-1^) and in FRAP assay (358 ± 0.02 mmol FeII/g dry extract). Radical scavenging activity of FME at 400 μg.ml^-1^ was comparable with α-tocopherol (40 μg.ml^-1^) and BHA (100 μg.ml^-1^) (*p* > 0.05). Other extracts did not have any antioxidant activity in comparison with *α-*tocopherol and BHA. Other extracts did not have any significant antioxidant activities.

**Table 1:** **Antioxidant activity and total phenolic content of different extracts from fruits and roots of *D. littoralis *** **subsp. *hyrcanicus***

	**DPPH**	**FRAP**	**Total phenol contents**
	**(μg.ml**^**-1**^**)**	**(mmol FeII/g dry extract)**	**(mg GAE/g dry extract)**
FEE	789.74	44.6 ± 0.2	25.13 ± 0.06
FME	145.93	358 ± 0.02	99.1 ± 0.08
FMWE	172.3	306 ± 0.08	41.35 ± 0.04
REE	>1000	78 ± 0.01	36.06 ± 0.01
RME	467.2	258 ± 0.30	32.12 ± 0.03
RMWE	269.75	214 ± 0.20	27.79 ± 0.01
Vitamin E	14.12	313 ± 0.01	-
BHA	7.8	880 ± 0.06	-

Key to extracts employed: FEE: Fruts Ethyl acetate Extract; FME: Fruits Methanol Extract; FMWE: Fruits Methanol–water Extract; REE: Roots Ethyl acetate Extract; RME: Roots Methanol Extract; RMWE: Roots Methanol–water Extract.

The results of antimicrobial assays are shown in Table 2. Among the extracts, only FME exhibited antimicrobial activity against all four microorganisms. FME showed better antimicrobial activity against *S. aureus* (MIC: 3.75 and MBC: 7.5 mg.ml^-1^), whereas it showed weak activity towards Gram negative bacteria.

**Table 2:** **Minimum inhibitory concentration (MIC) and minimum bactericidal concentration (MBC) of deferent extract of fruits and roots of *D. littoralis *** **subsp. *hyrcanicus***

**Microorganisms**	***Staphylococcus aureus***		***Escherichia coli***		***Pseudomonas aeruginosa***		***Candida albicans***	
	**MIC**	**MBC**	**MIC**	**MBC**	**MIC**	**MBC**	**MIC**	**MBC**
FEE	3.7	7.5	-	-	-	-	15.6	15.6
FME	3.7	7.5	>100	>100	62.5	>100	7.8	15.6
FMWE	-	-	-	-	-	-	-	-
REE	-	-	-	-	-	-	-	-
RME	>100	>100	-	-	-	-	-	-
RMWE	>100	>100	-	-	-	-	-	-

Note: MIC and MBC were determined by broth micro dilution method and expressed in mg.ml^-1^(W/V); Key to extracts employed: FEE: Fruits Ethyl acetate Extract; FME: Fruits Methanol Extract; FMWE: Fruits Methanol–water Extract; REE: Roots Ethyl acetate Extract; RME: Roots Methanol Extract; RMWE: Roots Methanol–water Extract.

Antiproliferative activity was determined in HepG2, MCF7, HT-29, and NIH-3T3 cells, shown in Table 3. Only FEE and root ethyl acetate extract (REE) showed toxicity on cancerous cell lines. FEE showed higher cytotoxicity on HepG2 and MCF7 (IC_50_ 185.01 ± 2.1 and 168 ± 1.5 μg.ml^-1^, respectively) than REE.

**Table 3:** **Cytotoxic activity of different extracts of fruits and roots *D. littoralis *** **subsp. *hyrcanicus *** **using MTT assay**

	**HepG2**	**MCF7**	**HT-29**	**NIH-3T3**
FEE	185.01 ± 2.1	168.41 ± 1.5	412.8 ± 1.3	149.48 ± 1.1
FME	- ^a^	-	-	-
FMWE	-	-	-	-
REE	219.58 ± 1.1	279.68 ± 2.6	351.26 ± 3.2	155.34 ± 1.3
RME	935.34 ± 2.4	-	-	-
RMWE	-	-	-	-
Methotrexate	-	0.16 ± 0.09	0.23 ± 0.02	0.24 ± 0.01

Notes: Results are expressed as IC_50_ values (μg.m.l^-1^), ^a^ inactive (IC_50_ > 1000); Key to cell Lines employed: HepG2(Hepatocellular carcinoma); MCF7 (breast carcinoma); HT29 (colon carcinoma); NIH-3T3 (Swiss embryo fibroblast). Key to extracts employed: FEE: Fruts Ethyl acetate Extract; FME: Fruits Methanol Extract; FMWE: Fruits Methanol–water Extract; REE: Roots Ethyl acetate Extract; RME: Roots Methanol Extract; RMWE: Roots Methanol–water Extract.

**Figure 1:**
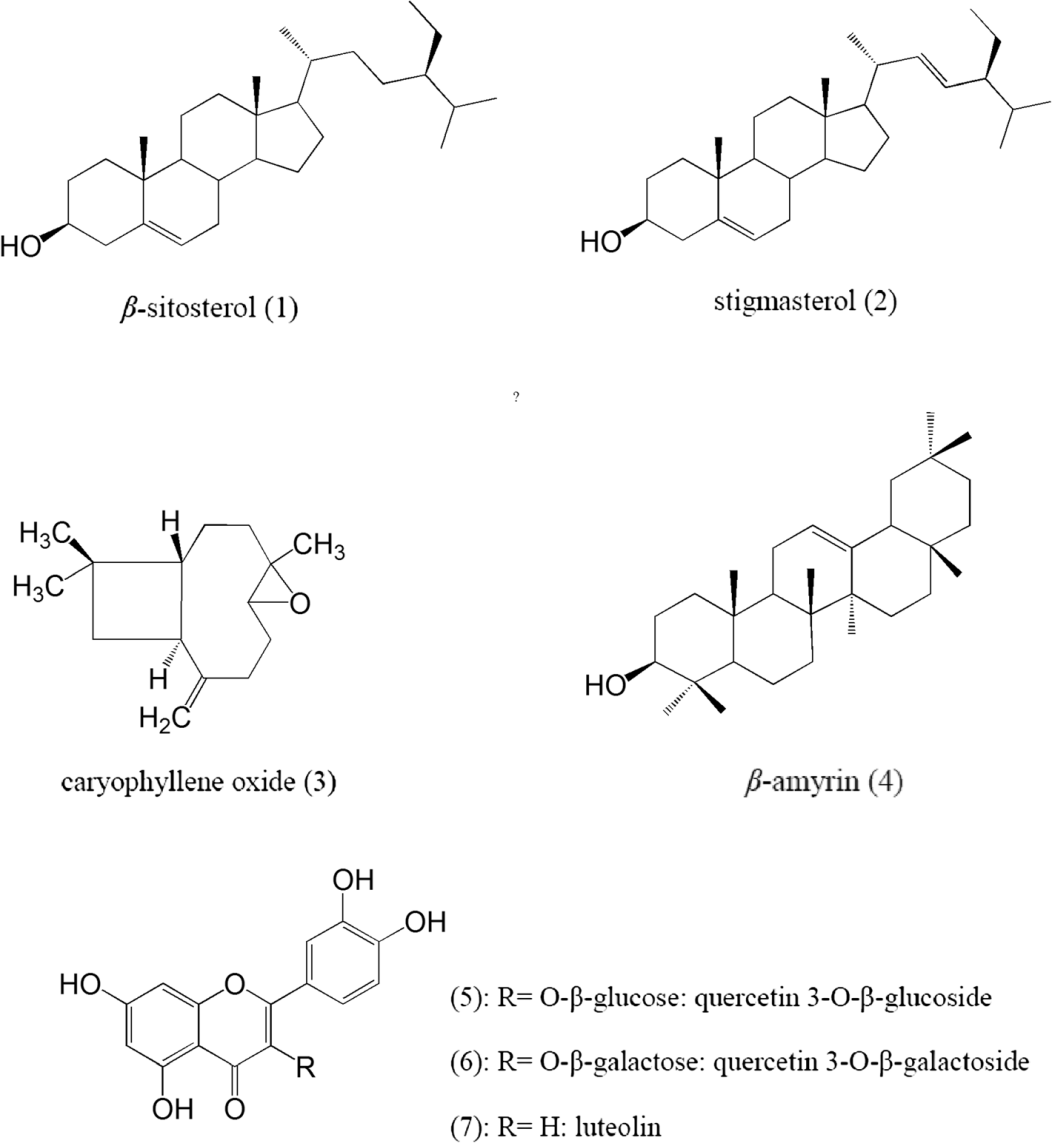
**Structures of compounds 1-7 isolated form *D. littoralis *** **Smith subsp. *hyrcanicus *** **Rech.f including β-sitosterol (1), stigmasterol (2), caryophyllene oxide (3), β-amyrin (4), quercetin 3-O-β-glucoside (5), quercetin 3-O-β-galactoside (6) and luteolin (7).**

The FEE and FME were used for isolation and purification of main components with different chromatography methods. From FEE four terpenoids including β-sitosterol (1), stigmasterol (2) caryophyllene oxide (3) and β-amyrin (4) were isolated. Three flavonoids including: quercetin 3-O-β-glucoside (5), quercetin 3-O-β-galactoside (6) and luteolin (7) were isolated from FME (Figure 1). Compounds 1–7 were identified by comparison of their spectroscopic data (^1^H-NMR, ^13^C-NMR) with those in the literature and authentic compounds from our laboratory [14-16]. Spectroscopic data of compounds 1-7 are provided in Additional file 1.

*β-*Amyrin is a pentacyclic triterpene with anti-inflammatory, antimicrobial, antifungal, antiviral and cytotoxic properties [17]. *β-*Amyrin isolated from MeOH extracts of *Byrsonima crassifolia* showed moderate antimicrobial activity against *S. aureus* and *C. albicans* (MIC: 0.5 and 1.02 mg.ml^-1^, respectively) [18]. The better activity of FEE against *S. aureus* and *C. albicans* seems to be due to the presence of *β-*amyrin. *β-*Sitosterol and stigmasterol are two of the most prevalent phytostrols in the plant kingdom [19]. *In vivo* investigation showed that oral consumption of stigmasterol inhibits the absorption of sterols and cholesterol from the intestinal tract and suppress the biosynthesis of cholesterol and bile acids in rats [19]. *β*-Sitosterol modulates the production of inflammatory cytokines, and reduces prostate enlargement [20]. It showed cytotoxic activity against colon carcinoma (COLO 320 DM), breast cancer and Bowes cell lines [21]. *β*-Caryophyllene oxide has shown cytotoxic activity against HepG2, AGS (human lung cancer cells), HeLa (human cervical adenocarcinoma cells), SNU-1 (human gastric cancer cell) and SNU-16 (human stomach cancer), with IC_50_ values of 3.95, 12.6, 13.55, 16.79, and 27.39 μM, respectively [22]. The cytotoxic activity of FEE against MCF7 and HepG2 seems to be due to the presence of *β*-sitosterol, *β*-amyrin and *β*-caryophyllene oxide.

In general, phenolic compounds possess antibacterial and antifungal properties [23]. Among them, flavonoids are well known for their antibacterial, antifungal, antiviral, antioxidant, anti-inflammatory activities [24]. The antioxidant activity of flavonoids is due to their capability as radical scavengers [25]. Many medicinal plants containing flavonoids have been reported for their antibacterial activity [23]. The high amount of total phenols and in particular quercetin 3-O-*β*-glucoside, quercetin 3-O-*β*-galactoside and luteolin are responsible for better antibacterial activity of FME.

## Conclusion

In this study, a screening of different extracts of *D. littoralis* Smith subsp. *hyrcanicus* Rech.f. was carried out for the first time. The presence of *β*-sitosterol, *β-*amyrin and *β* -caryophyllene oxide explained the cytotoxic activity of FEE in breast carcinoma and hepatocellular carcinoma cell lines. The high amount of phenolic compounds and flavonoids was responsible for the antioxidant and antimicrobial activity of FME. Based on these observations, FEE and FME can be good candidates for further *in vivo* biological studies and phytochemical investigations.

## Competing interests

No conflict of interest has been declared.

## Authors’ contributions

FY performed plant preparation, extraction, isolation, identification of plant substances, evaluated antimicrobial activity of extracts, advised on antioxidant and total phenol content method and drafted the manuscript. AR-G advised on separation of plant substances and identification of compounds. ZH carried out antioxidant assays, total phenol content and cytotoxic activity of the extracts. SN-O advised on cytotoxic activity of extract by MTT assay. MH-SS conceived the study and edited the manuscript. MA advised on NMR techniques of isolated compounds. FG contributed in antimicrobial assay and edited the manuscript. HJ advised antimicrobial activity of extracts. GR-A did the botanical studies and identified scientific name of the Plant, conceived the study and edited the manuscript. All authors read and approved the final manuscript.

## Supplementary Material

Additional file 1 **Spectroscopic data of compounds 1-7 isolated from *D. littoralis *** **Smith subsp. *hyrcanicus *** **Rech.f.:** Click here for file
