# Correction: Bioactive terpenoids and flavonoids from *Daucus littoralis* Smith subsp. *hyrcanicus* Rech.f, an endemic species of Iran

**DOI:** 10.1186/2008-2231-22-33

**Published:** 2014-04-02

**Authors:** Fatemeh Yousefbeyk, Ahmad Reza Gohari, Zeinabsadat Hashemighahderijani, Sayed Nasser Ostad, Mohamad Hossein Salehi Sourmaghi, Mohsen Amini, Fereshteh Golfakhrabadi, Hossein Jamalifar, Gholamreza Amin, Mohsen Amin

**Affiliations:** 1 Department of Pharmacognosy, Faculty of Pharmacy and Medicinal Plants, Research Centre, Tehran University of Medical Sciences, Tehran 14155-6451, Iran; 2 Department of Toxicology, Pharmacology and Nanotechnology Research Centre, Tehran University of Medical Sciences, Tehran 14155-6451, Iran; 3 Department of Medicinal Chemistry, Faculty of Pharmacy, Tehran University of Medical Sciences, Tehran 14155-6451, Iran; 4 Department of Drug and Food Control, Faculty of Pharmacy and Pharmaceutical Quality Assurance Research Centre, Tehran University of Medical Sciences, Tehran 14155-6451, Iran; 5 Department of Molecular Genetics, Faculty of Medicine, University of Toronto, 1 king’s college circle, Medical Sciences Building, Toronto, Ontario M5S 1A8, Canada

## Correction

After publication of this work [1] all authors noted they had failed to include Dr. Mohsen Amin in the authors’ list upon his contribution in antimicrobial assay and major editing and revising the manuscript. Author’s email, Author’s detail and Authors’ contribution is as following:

Author’s email

mohsen.amin@utoronto.ca

Authors’ detail

5 Department of Molecular Genetics, Faculty of Medicine, University of Toronto, 1 king’s college circle, Medical Sciences Building, Toronto, Ont. M5S 1A8, Canada

## Competing interests

The authors declare that they have no competing interests.

## Authors’ contributions

MAM contributed in antimicrobial assay and major editing and revising the manuscript.

## Acknowledgements

This investigation granted by research chancellor of Tehran University of Medical Sciences. Also we acknowledge Mr Amir Yousefbeyk for his assistance in collecting plants.
