# Supplementary material for: Bioactive Terpenoids and Flavonoids from Daucus littoralis Smith subsp. hyrcanicus Rech.f, an Endemic Species of Iran
Source: Daru. 2014 Jan 7;22(1):12. doi: 10.1186/2008-2231-22-12 (PMC4029373; doi:10.1186/2008-2231-22-12)
Supplement: Additional file 1 — Spectroscopic data of compounds 1-7 isolated from D. littoralis Smith subsp. hyrcanicus Rech.f.: [file 2008-2231-22-12-S1.doc]

**Additional file 1:**

Spectroscopic data of compounds 1-7 isolated from *D.* *littoralis* Smith subsp. *hyrcanicus* Rech.f.**:**

β-Sitosterol **(1****)**: ^1^H-NMR (500 MHz, CDCl_3_): δ (ppm), 0.68 (3H, s, H-18), 0.81 (3H, br s, H-26), 0.82 (3H, br s, H-27), 0.84 (3H, br s, H-24b), 0.92 (3H, d, J= 6.2 Hz, H-21), 1.03 (3H, s, H-19), 3.52 (1H, m, H-3), 5.35 (1H, m, H-6). ^13^C-NMR (125 MHz, CDCl_3_), δ (ppm): 11.8 (C-18), 11.9 (C-29), 18.8 (C-21), 19.0 (C-27), 19.4 (C-19), 19.8 (C-26), 21.1 (C-11), 23.0 (C-28), 24.3 (C-15), 26.0 (C-23), 28.3 (C-16), 29.1 (C-25), 31.6 (C-2), 31.8 (C-7), 31.9 (C-8), 33.9 (C-22), 36.1 (C-20), 36.5 (C-10), 37.2 (C-1), 39.7 (C-12), 42.3 (C-13), 45.8 (C-24), 50.1 (C-9), 56.0 (C-17), 56.8 (C-14), 71.8 (C-3), 121.7 (C-6), 140.7 (C-5)

Stigmasterol **(2):** ^1^H-NMR (500 MHz, CDCl_3_): δ (ppm), 0.68 (3H, s, H-18), 0.81 (3H, brs, H-26), 0.82 (3H, brs, H-27), 0.84 (3H, brs, H-24b), 0.94 (3H, d, J= 6.2 Hz, H-21), 1.01 (3H, s, H-19), 3.52 (1H, m, H-3), 5.04 (1H, m, H-23), 5.15 (1H, m, H-22), 5.35 (1H, m, H-6). ^13^C NMR(CDCl_3_, 125 MHz), δ (ppm): 11.8 (C-18), 11.9 (C-29), 18.8 (C-21), 19.0 (C-27), 19.4 (C-19), 19.8 (C-26), 21.1 (C-11), 23.0 (C-28), 24.3 (C-15), 26.0 (C-23), 28.3 (C-16), 29.1 (C-25), 31.6 (C-2), 31.8 (C-7), 31.9 (C-8), 33.9 (C-22), 36.1 (C-20), 36.5 (C-10), 37.2 (C-1), 39.7 (C-12), 42.3 (C-13), 45.8 (C-24), 50.1 (C-9), 56.0 (C-17), 56.8 (C-14), 71.8 (C-3), 121.7 (C-6), 129.2 (C-23), 138.3 (C-22), 140.7 (C-5)

Caryophyllene oxide **(3):** ^1^H-NMR (500 MHz, CDCl_3_), δ (ppm): 0.99 (3H, s, H-12), 1.01 (3H, s, H-13), 1.20 (3H, s, H-14), 1.76 (2H, m, H-3), 2.10 (2H, m, H-6), 2.25 (1H, m, H-7a), 2.34 (1H, m, H-7b), 2.61 (1H, m, H-9), 2.87 (1H, dd, J=10.4, 4 Hz, H-5), 4.86 (1H, brs, H-15a), 4.97 (1H, brs, H-15b).^13^C NMR (125 MHz, CDCl_3_), δ (ppm): 16.97 (C-12), 21.6 (C-13), 27.19 (C-10), 29.72 (C-2), 29.86 (C-14), 30.17 (C-3), 33.99 (C-11), 39.10 (C-6), 39.71(C-7), 48.70 (C-1), 50.72 (C-9), 59.8(C-4), 63.74 (C-5), 112.73 (C-15), 151.81 (C-8). EIMS m/z (rel. int.): 220 (M^+^, 3), 187(5), 138(12), 121(28), 109(37), 91(75), 79(100), 69(49).

β-Amyrin **(4):** ^1^H-NMR (500 Hz, CDCl_3_), δ (ppm): 0.79 (3H, s, H-24), 0.83 (3H, S, H-28), 0.88 (6H, s, H-29 and 30), 0.94 (3H, s, H-25), 0.97 (3H, s, H-26), 1.1 (3H, s, H-23), 3.2 (1H, dd, J= 11.2 Hz, H-3), 2.34 (1H, t, J= 7.2 Hz, H-9), 5.19 (1H, t, J= 3.45, H-12). ^13^ C- NMR (125 MHz, CDCl_3_), δ (ppm): 15.5 (C-24), 15.58 (C-25), 16.8 (C-26), 18.37 (C-6), 23.52 (C-11), 23.69 (C-30), 25.99 (C-27), 26.15 (C-15), 26.93 (C-2), 27.23 (C-16), 28.09 (C-23), 28.4 (C-28), 31.08 (C-20), 32.49 (C-17), 32.64 (C-7), 33.34 (C-29), 34.73 (C-21), 36.94 (C-10), 37.14 (C-22), 38.58 (C-1), 38.77 (C-4), 39.2 (C-8), 41.7 (C-14), 46.82 (C-19), 47.22 (C-18), 47.63 (C-9), 55.17 (C-5), 79.02 (C-3), 121.72 (C-12), 145.19 (C-13),

Quercetin 3-O-β-glucoside **(5):** ^1^H-NMR (500 Hz, DMSOd): δ (ppm): 6.19 (1H, d, J = 2.0 Hz, H-6), 6.40 (1H, d, J = 2.0 Hz, H-8), 7.53 (1H, d, J = 2.2 Hz, H-2'), 9.65 (1H, s, OH-3'), 6.83 (1H, d, J = 9.0 Hz, H-5'), 7.76 (1H, dd, J = 2.2, 9.0 Hz, H-6'), 5.47 (1H, d, J = 7.4 Hz, H-1′′), 3.1-3.60 (m, sugar protons); ^13^ C NMR (125 MHz, CDCl3), δ (ppm): 93.7 (C-8), 98.9 (C-6), 103.6 (C-10), 133.7 (C-3), 156.01 (C-9), 156.4 (C-2), 164.2 (C-7), 177.3 (C-4), 115.2 (C-2′) , 116.5 (C-5′), 121.4 (C-1′), 121.9 (C-6′), 144.9 (C-3′), 148.6 (C-4′), 61.1 (C-6′′), 70.1 (C-4′′), 74.1 (C-2′′), 76.5 (C-3′′), 77.6 (C-5′′), 101.1 (C-1′′).

Quercetin 3-O-β-galactoside **(6):** ^1^H-NMR (500 MHz, DMSO_d6_): δ (ppm): 6.20 (1H, d, J = 2.0 Hz, H-6), 6.40 (1H, d, J = 2.0 Hz, H-8), 7.52 (1H, d, J = 2.2 Hz, H-2'), 9.65 (1H, s, OH-3'), 6.82 (1H, d, J = 9.0 Hz, H-5'), 7.69 (1H, dd, J = 2.2, 9.0 Hz, H-6'), 5.38 (1H, d, J = 7.5 Hz, H-1′′), 3.2-3.6 (m, sugar protons);  ^13^ C- NMR (125 MHz, DMSO_d6_), δ (ppm): 93.5 (C-8), 98.6 (C-6), 103.6 (C-10), 133.7 (C-3), 156 (C-9), 156.3 (C-2), 164.2 (C-7), 177.2 (C-4), 115.2(C-2′), 116.3 (C-5′), 121.3 (C-1′), 121.9 (C-6′), 144.7 (C-3′), 148.6 (C-4′), 60.1 (C-6′′), 67.9 (C-4′′), 71.2 (C-2′′), 73.2 (C-3′′), 75.8 (C-5′′), 102 (C-1′′)

Luteolin (7): ^1^H-NMR (500 MHz, DMSO_d6_): δ (ppm): 6.18 (1H, d, J=1.8, H-6), 6.43 (1H, d, J=1.8, H-8), 6.6 (1H, s, H-3), 6.88 (1H, d, J=8.2, H-5′), 7.38 (1H, dd, H-6′), 12.96 (OH-5). UVλ max (MeOH) nm: 245(sh), 255, 267, 291(sh), 350.
